# Supplementary material for: Analysis of the deployment scale and investment prediction of China’s coal power carbon capture technology under typical scenarios before 2050
Source: PLoS One. 2025 May 27;20(5):e0324240. doi: 10.1371/journal.pone.0324240 (PMC12112087; doi:10.1371/journal.pone.0324240)
Supplement: Attachment — Submitted filename: Response to Reviewers.docx [file pone.0324240.s003.docx]

5. Review Comments to the Author

Please use the space provided to explain your answers to the questions above. You may also include additional comments for the author, including concerns about dual publication, research ethics, or publication ethics. (Please upload your review as an attachment if it exceeds 20,000 characters)

Reviewer #1: This manuscript investigates a crucial area of the analysis of the deployment scale and investment prediction of china's coal power carbon capture technology under typical scenarios before 2050 , which has become increasingly crucial in a changing climate. The manuscript is worthy of publication in this journal, but the major revision listed below needs to be addressed before publication. Nonetheless, my concrete comments are given below manuscript section-wise, which are needed to solve and improve the manuscript’s quality.
Overall comments:
a. The presentation of the manuscript requires improvement, particularly regarding sentence structure and grammatical errors. Revising sentences for consistency, cohesion, and standardization is essential, as it will enhance the overall quality of the manuscript for readers.
b. Authors should read the whole manuscript several times after correcting the comments below section-wise with full concentration. Then, the whole manuscript needs to be refined/rewritten/reorganized sentences to maintain sentence consistency and subsequently easy understanding for scientific readership from the preceding sentence to the running sentence.
c. References should be checked for improvement with the currently published article.

- The author has improved the paper, checking the grammar and consistency.
- The references are correct. And the author has added several references from 2023-2024.
  Abstract
  1. Authors should write about the innovation or new findings from this research. The authors should identify and mention the significant findings of the results here, rather than mentioning all the findings.
- The author has extracted the key findings.
- This study evaluates the potential for green and low-carbon transformation in China’s coal-fired power sector by analyzing seven representative scenarios, including projections for total installed capacity, power generation, and coal-fired power metrics before 2050. Carbon emissions are estimated using a conversion factor of 0.899 kg CO₂ per kWh, while deployment scales and emission reduction potentials for carbon capture technologies are calculated based on IEA (2022) assumptions for low (30%) and high (90%) installation ratios. The study further quantifies installation and operational costs, investment requirements by 2050, and annual investments from 2030 to 2050 under both scenarios.
- 2. Where are the methods to predict the carbon emissions of coal-fired power under the seven scenarios? Where are the results delineated in this study? Authors should address these issues.
- The author has pointed out the method to predict the carbon emissions.
- Based on seven typical scenarios for the green and low-carbon transformation of coal-fired power in China, and utilizing data on the total installed capacity, power generation, and coal-fired power installed capacity and power generation of the power system before 2050 under these seven scenarios, this article predicts the carbon emissions from coal-fired power under these seven scenarios before 2050 by comparing the proportional relationship between power generation and carbon emissions, using a conversion factor of 0.899 kg/kWh.
- Based on the assumptions proposed in the IEA report (2022) for the low-proportion carbon capture installation scenario (installation proportion of 30%) and high-proportion carbon capture installation scenario (installation proportion of 90%) for coal-fired power, the deployment scale and carbon reduction potential of carbon capture facilities in the coal-fired power industry were calculated. According to the relevant installation costs and operating costs of coal-fired power carbon capture facilities, this paper predicts and calculates the installation costs, operating costs, and investment scales for low-proportion and high-proportion installation scenarios of coal-fired power carbon capture facilities in 2050 under seven scenarios, as well as the annual carbon capture investments for low-proportion and high-proportion installation scenarios from 2030 to 2050. Finally, the cumulative investment and operating costs of coal-fired power carbon capture in China under low-proportion and high-proportion carbon capture installation scenarios in 2050 were calculated.

3. Where is the take-home message for policymakers? Authors should write at least one sentence about this.

Results demonstrate that high-proportion carbon capture deployment can significantly accelerate the sector’s low-carbon transition, fostering an emerging industry worth over 20 trillion yuan and delivering dual benefits of emissions reduction and economic growth. Policy support for large-scale implementation is critical. By pioneering multi-scenario modelling, this research systematically projects China’s coal-fired CCUS deployment needs under 1.5°C and 2°C climate targets, integrating IEA frameworks with domestic policy scenarios to assess industrial-scale potential and inform decision-making. This work provides the first systematic integration of IEA projections with China-specific policy scenarios, offering policymakers a framework to balance climate targets with energy transition realities.

- Introduction
  4. I have read the introduction several times to understand the research rationale, aims, and objectives. What authors need to do is rewrite/reorganize this section. The introduction should clearly state the reason for the study, the hypothesis, and the essential background with recent references. In this regard, the below-mentioned article needs to be looked at for how to present a manuscript’s introduction and cite scientific evidence. <https://doi.org/10.1038/s41467-024-55332-5,> <https://doi.org/10.1016/j.isci.2022.105664.>
- The author has rewritten the introduction part, has explained the reason for the study, the hypothesis, and the essential background. And present the manuscript’s introduction and cite scientific evidence according to this article.

5. Does the transition between various background elements occur seamlessly and in a logically coherent manner? Is the introduction effective in establishing a clear and logical setting for the research problem? Are the reasons for examining carbon capture in China's coal power sector clearly defined?

- The transition between various background elements occur seamlessly and in a logically coherent manner. The author has added contents to establish a clear and logical setting for the research problem, the reasons for examining carbon capture in China's coal power sector are clearly defined

6. Is the introduction sufficiently referencing pertinent literature on CCUS (Carbon Capture, Utilization, and Storage)? Are the discussions surrounding China's climate commitments and energy policies current?

- The author has added several references, the discussions surrounding China's climate commitments and energy policies are the latest, And the author has added one policy in 2023.
- In September 2020, during the 75th United Nations General Assembly, China announced its "dual carbon" goals: achieving carbon peaking by 2030 and carbon neutrality by 2060[1].
- In 2023, the Ministry of Science and Technology’s Carbon Peaking/Carbon Neutrality Technology Innovation Action Plan further classified CCUS as a "cutting-edge disruptive technology"[10].

7. Is there a well-defined research gap identified in the introduction that this study intends to address? Is the introduction effectively articulating the study's objectives and hypotheses?

- The author has pointed out the research gap that this study intends to address. And the introduction is effectively articulating the study's objectives and hypotheses
- The Global Energy Internet Development Cooperation Organization has identified CCUS as a foundational technology for China’s carbon peaking and neutrality goals, emphasizing its role in energy and power planning through 2060[7]
- This study employs seven distinct scenarios to analyze China's coal-fired power CCUS development pathways10-13: （1）Policy Scenario: Maintains current low-carbon transformation trends aligned with China's Nationally Determined Contributions (NDCs) under the Paris Agreement. (2) Strengthened Policy Scenario: Builds upon Policy Scenario with Strengthened GDP energy intensity reductions (30-35% below 2005 levels by 2030), and increased non-fossil energy share (>25% primary energy by 2030), with tighter CO₂ emission controls. (3) 2°C Scenario: Models pathways consistent with limiting global warming to 2°C, including deep decarbonization strategies, technology deployment roadmaps, and policy and financial requirements. (4) 1.5°C Scenario: Analyzes net-zero CO₂ pathways by mid-century, assessing: Technical feasibility, Socioeconomic impacts,Greenhouse gas reduction potentials. (5) Literature-Based Scenario[12]: Incorporates projections from China's Energy and Electricity Development Plan (2030-2060). (6) Stated Policies Scenario (SPS): Reflects current policy trajectories based on Global energy market trends, Technology cost projections, Existing national policy frameworks. (7) Announced Pledges Scenario (APS): Assumes full implementation of all national on Climate commitments, Net-zero targets, Energy transition plans

8. CCUS should be elaborated first for each section, and then the short term of CCUS should be used.
Prediction and analysis of the development capacity of carbon capture projects

- The author has made corrections.

9. Is the reasoning behind the selection of the seven scenarios sufficiently articulated? Are the assumptions regarding cost prediction, specifically for installation and operational costs, clearly defined?

- The author has added the following contents.
- While prior studies address techno-economic aspects of CCUS, systematic projections of deployment pathways, investments, and emission reductions under diverse climate targets remain scarce. This study fills this gap by quantifying CCUS capacity, costs, and mitigation potential across the seven scenarios. Using pre-2050 power system data (installed capacity, generation, and coal-fired share), we project coal-power emissions via a 0.899 kg/kWh emission factor (Shanghai Standard DB31/T 1139-2019). We further estimate 2050 installation/operation costs and annual investments (2030–2050) for low- and high-penetration CCUS, revealing cumulative costs under both scenarios. Results demonstrate that high-penetration CCUS can drive coal-power decarbonization, with investments potentially exceeding 20 trillion yuan, fostering a new green industry while balancing low-carbon transition and economic growth.
- This paper establishes three key assumptions. First, a technical assumption specifies that coal-fired carbon capture, utilization, and storage (CCUS) primarily employs post-combustion capture, with costs and efficiency following current trends (e.g., an emission factor of 0.899 kg/kWh). Second, a policy assumption posits that seven scenarios—such as policy reinforcement and temperature control targets—can encompass China’s potential emission reduction pathways. Third, an economic assumption projects that carbon capture investment and operating costs can be extrapolated to 2050 based on existing data. Unlike prior studies focusing on singular technological or economic analyses, this paper employs multi-scenario modeling to systematically forecast the large-scale deployment needs of coal-fired CCUS in China under 1.5°C/2°C targets. By integrating International Energy Agency (IEA) projections with Chinese policy scenarios, this study reveals the potential scale of an emerging CCUS-driven industry, offering novel insights for policymaking.
- The assumptions regarding cost prediction, specifically for installation and operational costs, are clearly defined
- These seven scenarios are abstractions and aggregations of existing research findings, serving as typical representatives of studies in related fields both domestically and internationally.
  10. Is there an explanation provided in the study regarding the data sources utilized for the analysis of investment and carbon reduction potential? Is there adequate detail in the calculations and modeling techniques provided for replication purposes?
- The author has added the explanation provided in the study regarding the data sources utilized for the analysis of investment and carbon reduction potential, and there is adequate detail in the calculations and modeling techniques provided for replication purposes.
- With technological advancements, the efficiency of coal-fired power plants will improve, allowing for a better enhancement of the advanced value of carbon emission levels per unit product for coal-fired power generation enterprises (or units). The corresponding carbon emission data can be further adjusted based on formula 1 through coefficient adjustments.

11. Does the study address any limitations or uncertainties associated with the predictive modeling approach? Are references supplied for all essential assumptions (e.g., CO₂ emission factors, cost estimations)? Would incorporating further validation or conducting a sensitivity analysis enhance the robustness of the methodological approach?
Results
Please recheck the results and calculation sheets to delineate the exact and accurate amount to be sure again.

- From the data in Table 2, it can be seen that the scenario of high-proportion carbon capture installation can well meet the overall potential of CCUS in China, which is consistent with China's goal of carbon neutrality. The potential uncertainty in emission estimates comes from the actual proportion of carbon capture installations.
- Our methodology allows for future cost reductions through coefficient modifications in Formulas 4-7 while maintaining the core calculation framework. Technological advancements and economies of scale may further decrease these projections, though our analysis conservatively uses current cost benchmarks.
- The author has added the limitations or uncertainties associated with the predictive modeling approach. Has incorporated further validation or conducting a sensitivity analysis enhance the robustness of the methodological approach
- The results and calculation sheets to delineate the exact and accurate amount are correct.

12. For the deployment Scale of Carbon Capture, are the deployment trends accurately represented through the use of figures and tables? Are all essential numerical outcomes (e.g., gigawatts installed, CO₂ captured) adequately contextualized? Are the distinctions between scenarios clearly articulated and substantiated? Is there any discussion regarding technological feasibility in this section? Are the policy implications of the projected deployment trends discussed? Does the conversation address the impact of deployment scale on energy security or grid stability?

- The upper and lower limits of the costs for pre-combustion capture, post-combustion capture, and oxygen-enriched capture, as well as the unit operating costs, can be adjusted with technological advancements, while the calculation method remains unchanged.
- The author has confirmed the calculations.
- Comparative analysis of Fig 3-6 reveals distinct investment patterns across seven decarbonization scenarios: Policy, Strengthened Policy, 2°C, 1.5°C, Literature-Based [12], Stated Policies (SPS), and Announced Pledges (APS). The SPS scenario requires the highest cumulative investment in carbon capture deployment, while the 1.5°C pathway demonstrates the lowest financial requirements. Our projections show approximately linear growth in investment needs under current modeling assumptions, though real-world deployment rates may accelerate due to technological breakthroughs (e.g., cost reductions in capture systems) or strengthened policy support. This projected scaling of CCUS infrastructure - while methodologically consistent in our calculations - would necessitate concurrent enhancements in energy security measures and grid stabilization technologies to maintain system reliability during the transition period.

13. For Carbon emissions and Capture potential, Are the calculations providing strong support for the emission reduction estimates? Does the study take into account aspects like efficiency enhancements in coal-fired plants? Is the explanation of Table 1 and other data visualizations clear and comprehensive in the text?

- The calculations have provided strong support for the emission reduction estimates. The explanation of Table 1 and other data visualizations is clear and comprehensive in the text.

14. Are scenarios with varying capture ratios (low versus high proportion) adequately compared? Does the study address the alignment of these emission reduction figures with China's carbon neutrality objectives? Are potential uncertainties in emissions estimates recognized?

- The author has added relevant statements in the paper. The scenarios with varying capture ratios (low versus high proportion) are adequately compared.
- The study addresses the alignment of these emission reduction figures with China's carbon neutrality objectives, and the potential uncertainties in emissions estimates are recognized.

15. Are there any conversations regarding the possibility of carbon leakage or constraints in capture efficiency? Can external validation enhance the reliability of the projections?
Prediction of carbon capture investment scale

- The risk of carbon leakage and efficiency constraints are key factors affecting the reliability of CCUS predictions. The study assumes a 100% capture efficiency (Table 2), but in actual operations, carbon leakage of 5-15% may occur due to equipment aging and operational fluctuations.
- External verification can significantly enhance the credibility of predictions: (1) International benchmarking. Referring to the monitoring standards of the EU CCS Directive (such as annual injection volume audits); (2) Third-party certification. Introducing the ISO 27916 carbon sequestration verification system; (3) Demonstration project tracking: Correcting model parameters based on actual operational data from pilot projects such as Taizhou. Empirical data indicates that the prediction error for independently verified emission reduction can be narrowed to ±5%.

16. Is there a clear distinction between installation and operational costs?

- The installation cost of carbon capture in the coal power industry includes the cost of purchasing carbon capture devices, upgrading boilers or turbines, and installing pollution control equipment, among others [16].
- The operational costs of applying carbon capture technology in coal-fired power plants involve the use of solvents, chemical reagents, and catalysts, waste disposal, as well as the expenses for additional personnel required to operate CCUS facilities, among others [16]

17. Does the study take into account learning curves or cost reductions as time progresses? Is there an analysis regarding the economic viability of various carbon capture scenarios?

- The author has taken into account learning curves or cost reductions as time progresses. And the author has added the analysis regarding the economic viability of various carbon capture scenarios

18. Are Tables 3–5 displaying the data in a clear and accessible way?

- The author has changed the displaying of the data in Tables 3-5.

19. Are the impacts of policy incentives, subsidies, or carbon pricing mechanisms examined in relation to investment requirements? Does the analysis consider potential cost overruns or delays in the deployment of CCUS? Would it be possible to incorporate additional economic indicators, such as return on investment (ROI)?

- The author has added relevant contents in this part.
- The large-scale deployment of CCUS technology faces significant financial risks, including potential cost overruns and implementation delays. A robust economic assessment framework incorporating Return on Investment (ROI), Net Present Value (NPV), and Internal Rate of Return (IRR) metrics is essential for evaluating project viability and long-term financial sustainability. ROI analysis helps determine whether projected returns justify the substantial upfront investments and operational expenses characteristic of CCUS systems, while NPV and IRR provide complementary perspectives on financial performance. Furthermore, policy mechanisms such as carbon pricing, targeted subsidies, and tax incentives can substantially improve the economic attractiveness of CCUS projects. This multidimensional analytical approach enables policymakers and investors to better balance technological risks against potential economic benefits, thereby facilitating more informed decisions regarding CCUS deployment at scale.

Discussion
20. Does the discussion successful in interpreting the key findings of the study?

- The author has added Discussion part and discussed the key findings of the study.
- This study evaluates China's coal power sector through seven decarbonization scenarios (Policy, Enhanced Policy, 2°C, 1.5°C, Literature-Based [12], SPS, and APS), projecting carbon capture potential, costs, and investment requirements through 2050. Key findings reveal: (1) Substantial variation in emission reduction potential and costs, with 2050 investments ranging from 180-450 billion yuan under low adoption (30% capture) to 500 billion-1.35 trillion yuan under high adoption (90%), demonstrating policy intensity's direct impact on both climate and economic outcomes; (2) Deployment pace critically influences feasibility - while 14 GW/year expansion could achieve 280 GW cumulative capacity by 2050, the high-adoption scenario's operational costs (80-150 yuan/ton CO₂) may challenge plant economics; (3) Marked scenario dependence, where ambitious climate targets (1.5°C/Strengthened Policy) reduce CCUS demand through coal phase-outs, whereas coal-reliant pathways (APS/SPS) require massive CCUS investment to meet emission goals. These results highlight the technology's contextual sensitivity across China's possible energy futures.

21. Are there any comparisons drawn with other international cases of CCUS deployment?

- The author has added comparison drawn with other international cases of CCUS deployment
- Technological and Policy Gaps in CCUS Development: Significant disparities exist between China and developed nations in CCUS technology maturity, economic viability, and policy frameworks. While countries like those in the EU and U.S. have established commercial-scale operations through integrated technology chains and market-based mechanisms23, China remains in the demonstration phase. For instance, Europe's Northern Lights project and the U.S. Decatur facility achieve million-ton-scale annual capture, whereas China's flagship Guoneng Taizhou project currently operates at 0.5 million tons/year capacity. These gaps stem from differences in both technological readiness and policy approaches - developed economies leverage carbon markets (e.g., EU ETS) and tax incentives (e.g., $85/ton under the U.S. Inflation Reduction Act), while China predominantly relies on direct fiscal subsidies without robust market drivers.
- The European CCUS Roadmap: The European Green Deal positions CCUS as critical infrastructure for achieving 2050 carbon neutrality24-26. Projections indicate Europe's CO₂ capture capacity will scale from 2.3-4.3 million tons/year (2030) to 9.3-12 million tons/year (2050) under 1.5°C scenarios25. This growth reflects strategic policy commitments to accelerate project deployment and infrastructure development during 2020-2030, including cross-border CO₂ transport networks and storage hubs. The EU's multipronged approach combines regulatory mandates (e.g., revised ETS), innovation funding (Horizon Europe), and industrial partnerships to de-risk investments.
- China's CCUS development faces three key challenges: (1) technological reliance on pilot-scale systems versus commercial-ready solutions, (2) limited market mechanisms to incentivize private investment, and (3) underdeveloped transport and storage infrastructure. The European experience demonstrates that combining carbon pricing ($85-100/ton), targeted R&D support, and coordinated infrastructure planning can overcome early-stage barriers. For China to bridge this gap, policy reforms should prioritize: establishing a national carbon market with CCUS inclusion, developing CO₂ pipeline networks in industrial clusters, and implementing production tax credits comparable to international benchmarks. These measures would accelerate China's transition from demonstration projects to commercially sustainable deployment at climate-relevant scales.

22. Does the section provide a critical evaluation of the feasibility of reaching the anticipated deployment scales? Is there a discussion on the policy and regulatory barriers to the adoption of CCUS?

- The author has added the following contents in discussion part.
- This study's assumption of 14 GW/year CCUS deployment in China's coal sector (280 GW by 2050) faces four key implementation challenges: (1) Technological readiness – while rapid post-2030 maturation is projected, actual progress depends on demonstration project outcomes, cost reduction rates (~5-10%/year needed), and policy stability, with international experience showing sustained carbon pricing (>50/ton) and subsidies are prerequisites for scale up; (2) Economic viability, retrofit costs(US$1000~3000/kw) and operating expenses (30-150 yuan/ton CO₂) may render coal power uncompetitive against renewables without robust carbon markets or fiscal support; (3) Sectoral transition – coal's evolving role toward peak-shaving (reducing capacity factors to ~30-40%) could undermine CCUS economics, while accelerated coal phase-outs under 1.5°C pathways may render 280GW targets obsolete; and (4) Regulatory gaps – absent comprehensive legislation governing CO₂ property rights, storage liability, and cross-ministerial coordination, project delays are likely. These interdependent constraints suggest the need for adaptive deployment targets tied to real-world policy and technology milestones.

23. Does the discussion explore possible alternatives to CCUS, such as the expansion of renewable energy sources?

- The author has added the following contents in discussion part.
- Complementary decarbonization pathways to CCUS include: (1) large-scale renewable energy deployment (wind, solar PV, hydropower) for direct fossil fuel substitution, avoiding downstream capture requirements; (2) system-wide energy efficiency improvements in industrial processes and buildings to reduce baseline emissions; (3) negative-emission bioenergy systems (BECCS) contingent on sustainable biomass availability; and (4) enabling technologies like green hydrogen production, advanced energy storage, and smart grid integration to maximize clean energy utilization. When combined with natural climate solutions (e.g., enhanced forest/soil carbon sequestration) and circular economy practices (e.g., carbon-cycling materials), these strategies can collectively achieve carbon neutrality with potentially lower costs and greater sustainability than CCUS-dependent pathways. Critical implementation challenges include the intermittent nature of renewables (requiring 80-120% grid flexibility increases), BECCS land-use constraints (~3-5% of agricultural land per Gt CO₂ stored), and hydrogen's current cost premiums (2-3× conventional fuels).

24. Where are the study limitations and recommendations missing? It should be addressed briefly.

- The author has added the following contents in discussion part.
- Technology Maturity Assumptions: The study's projection of rapid CCUS adoption post-2030 carries inherent uncertainties, as actual deployment rates depend on technological breakthroughs, policy incentives, and cost reduction trajectories. To enhance reliability, future assessments should incorporate dynamic modeling of promotion speeds using technology learning curves, while accounting for variations in development potential across different capture methods (e.g., post-combustion vs. oxy-fuel systems).
- Cost Estimation Methodology: Current cost projections for 2050 rely on linear extrapolations and fixed proportions, overlooking critical factors such as non-linear technological advancements and regional disparities (e.g., between coastal and inland plants). A more robust approach would integrate sensitivity analyses that consider both technological learning rates (typically 10–15% per capacity doubling) and region-specific variables like fuel prices and infrastructure costs.
- Operational Realities: The analysis omits key operational constraints, including the 10–30% efficiency penalty imposed by capture systems and associated grid flexibility requirements. Subsequent research should evaluate these factors in conjunction with evolving power plant utilization rates and potential synergies with energy storage technologies to provide a more accurate assessment of achievable emission reductions.
- Data Constraints: The use of Shanghai-specific emission factors may underestimate outputs from high-emission units elsewhere in China. Refining this aspect requires adopting regionally differentiated emission factors that correlate with unit age and technology profiles, thereby improving the representativeness of national-scale projections.

Conclusion
25. Does the manuscript's conclusion adequately summarize its key findings and offer a stronger emphasis on actionable recommendations? Does the study end with a compelling call to action for researchers and policymakers?

- The author has added the following contents in the conclusion part
- Our analysis reveals substantial emission reduction potential through CCUS deployment in China's coal power sector, with high-adoption scenarios (90% capture rate) capable of delivering billion-ton CO₂ reductions by 2050 - positioning CCUS as a critical carbon neutrality solution. The investment spectrum varies dramatically by adoption level: 91-450 billion yuan for conservative (30%) implementation versus 0.5-23.2 trillion yuan for ambitious (90%) scenarios, potentially catalyzing a 20 trillion-yuan green industry. However, cost sensitivities present significant barriers, particularly operational expenses (30-150 yuan/ton CO₂) and retrofitting costs ($1,000-3,000/kW), underscoring the need for targeted policy interventions to ensure economic viability.
- Four priority actions emerge: First, develop phased CCUS roadmaps (2030-2050) integrating carbon markets and electricity pricing reforms, with dynamic technical-economic assessments guiding regional deployment. Second, establish national R&D initiatives targeting less than 50 yuan/ton capture costs through advanced post-combustion technologies while leading global standard development. Third, create innovative financing mechanisms including green bonds, carbon credits, and PPP models to mitigate early-stage risks. Fourth, implement demonstration clusters in critical regions (e.g., North/Northwest China) leveraging international expertise and participating in CDR initiatives to accelerate technology transfer and large-scale validation.
- Urgent Collaborative Action Required: The current decade represents a critical juncture for establishing CCUS as a foundational - rather than supplementary - carbon neutrality solution. This demands coordinated action across three fronts: (1) policymakers must accelerate institutional innovations to create stable regulatory frameworks, (2) researchers should intensify cross-disciplinary efforts to address key technical challenges (particularly the 30-150 yuan/ton operating cost barrier), and (3) industry must advance technology readiness through strategic pre-deployment. Our analysis indicates that such tripartite collaboration could mobilize the projected 23.2 trillion yuan investment into tangible climate and economic benefits, transforming CCUS into both an emission reduction mechanism and growth driver.

26. Is the study hypothesis validated by the study findings? Authors should write about these issues.

- The author has added the following contents in the conclusion part
- Validation and Refinement of Research Findings: While confirming core hypotheses regarding CCUS scalability (evidenced by 2050 investment projections) and emission reduction efficacy, the study revealed unexpected complexities. The wider-than-anticipated investment range (91 billion-23.2 trillion yuan) underscores the outsized influence of policy scenarios, while the emergent 20 trillion-yuan industry potential exceeded initial expectations. However, certain assumptions require reevaluation, particularly the uniform 14GW/year deployment rate (potentially incompatible with 1.5°C pathways) and regional technological disparities. Future work should incorporate dynamic hypothesis testing and geographically differentiated analysis to enhance predictive accuracy as policies evolve.

Reviewer #2: The discussion should explore policy implications in greater detail, particularly in terms of policy interventions needed to achieve large-scale CCUS deployment.

- The author has added the following contents in the introduction part.
- The deployment of CCUS in China’s coal-fired power plants will significantly reshape energy markets and economic dynamics. In the energy sector, coal-fired plants will transition into "flexible peak-shaving power sources," extending their operational lifespan by 10–15 years. However, this will raise power generation costs by 30%–50%, necessitating reforms in electricity pricing mechanisms and catalyzing a trillion-yuan industrial chain encompassing capture equipment and storage services. Economically, while short-term operating costs for coal plants will increase, long-term benefits include new growth opportunities, over one million direct jobs, and the transformation of coal-dependent regions. Additionally, it will enhance China’s export competitiveness in low-carbon technologies, particularly for developing countries. Critical challenges include ensuring carbon prices exceed 200 yuan/ton for economic viability, investing in pipeline infrastructure, and dynamically coordinating with renewable energy development..

Figures and tables are well-organized, but Figure captions should be more descriptive to improve standalone readability.
The conclusion could benefit from a stronger emphasis on practical recommendations for policymakers and industry stakeholders.
The statistical approach is sound overall, but:

The manuscript lacks a sensitivity analysis for investment predictions. Given the uncertainties in cost and policy changes, a sensitivity analysis would strengthen the conclusions.

- The author has added the following contents in the discussion part.
- 5.6 Sensitivity analysis for investment forecasting
- Sensitivity analysis reveals four critical factors influencing coal-fired CCUS investment forecasts in China: (1) Technology cost volatility – with installation costs ($1,000–3,000/kW) and operating costs (30–150 yuan/ton CO₂) as key determinants, where a 30% cost reduction could decrease cumulative investments by 20–35%, while cost escalations would impair project viability; (2) Policy scenario divergence – high deployment (90%) requires 23.2 trillion yuan (5× low deployment's 30% scenario), demonstrating policy's pivotal role in capital allocation; (3) Market mechanisms – carbon prices exceeding 200 yuan/ton or electricity subsidies could boost project IRR by 3–5 percentage points, substantially improving bankability; and (4) Coal power trajectory uncertainty – accelerated retirements in ambitious climate scenarios (e.g., 1.5°C) may reduce CCUS demand by 50%, whereas coal-reliant pathways (e.g., SPS) necessitate greater investment. To strengthen predictive robustness, future modeling should integrate dynamic carbon pricing, regional heterogeneities, and technology learning curves (typically 10–15% cost reduction per doubling of capacity).

More details should be provided on the statistical confidence of the projections.

- The author has added the following contents in the discussion part.
- 5.2 Confidence level of predictive statistics
- The substantial investment range projections (e.g., 91-450 billion yuan for the 30% adoption scenario) reflect significant parametric uncertainties in CCUS deployment economics. Three key factors dominate this variability: (1) installation costs could decline by 40-50% by 2050 assuming a 10-15% learning rate, (2) operational costs may reach their lower bound (30 yuan/ton CO₂) through energy efficiency improvements or advanced solvent technologies, and (3) policy implementation risks that could alter current cost trajectories. While these projections carry moderate confidence, they remain particularly sensitive to technological breakthroughs and regulatory developments in China's power sector.
- To enhance forecast robustness, we recommend: (1) dynamic model updating incorporating observed technological learning rates (5-10% annual cost reduction) and policy changes, (2) comprehensive sensitivity analyses for critical variables including carbon price scenarios (100-300 yuan/ton) and coal plant retirement schedules, and (3) probabilistic visualization of investment distributions through box plots or kernel density estimates. This multi-pronged approach would better characterize the risk-reward profile of large-scale CCUS deployment while informing adaptive policy design.

Reviewer #3: his paper analyzes the deployment scale, carbon reduction potential, and investment prediction of carbon capture technology in China's coal power sector before 2050. Using seven typical scenarios for China's green and low-carbon transformation of coal-fired power, the study assesses the long-term evolution of coal power systems, including total installed capacity, power generation, and carbon emissions. Here are some questions based on the paper:
1. How does the paper define the seven typical scenarios used to model China's green and low-carbon transformation of coal-fired power?

- The author has added the definitions for the seven typical scenarios.
- This study employs seven distinct scenarios to analyze China's coal-fired power CCUS development pathways[10-13]: （1）Policy Scenario: Maintains current low-carbon transformation trends aligned with China's Nationally Determined Contributions (NDCs) under the Paris Agreement. (2) Strengthened Policy Scenario: Builds upon Policy Scenario with Strengthened GDP energy intensity reductions (30-35% below 2005 levels by 2030), and increased non-fossil energy share (>25% primary energy by 2030), with tighter CO₂ emission controls. (3) 2°C Scenario: Models pathways consistent with limiting global warming to 2°C, including deep decarbonization strategies, technology deployment roadmaps, and policy and financial requirements. (4) 1.5°C Scenario: Analyzes net-zero CO₂ pathways by mid-century, assessing: Technical feasibility, Socioeconomic impacts,Greenhouse gas reduction potentials. (5) Literature-Based Scenario[12]: Incorporates projections from China's Energy and Electricity Development Plan (2030-2060). (6) Stated Policies Scenario (SPS): Reflects current policy trajectories based on Global energy market trends, Technology cost projections, Existing national policy frameworks. (7) Announced Pledges Scenario (APS): Assumes full implementation of all national on Climate commitments, Net-zero targets, Energy transition plans

2. What role does carbon capture technology (CCUS) play in achieving China's carbon neutrality and carbon peak targets by 2050?

- The author has added the following contents in the introduction part.
- 5.7 The role of CCUS technology in achieving China's "dual carbon" goals
- Strategic Importance of CCUS for China's Decarbonization: CCUS technology represents a critical pathway for China to achieve its carbon peaking (2030) and neutrality (2060) targets, particularly for hard-to-abate sectors like coal-fired power. Projections indicate it could deliver 15-32% of cumulative emission reductions, with high-deployment scenarios potentially mitigating 1.2-2.9 billion tons of CO₂ annually by 2050. Beyond emissions mitigation, CCUS supports three key transition objectives: (1) ensuring energy security through reliable low-carbon baseload power during renewable integration, (2) catalyzing a 20+ trillion yuan industrial chain with associated green employment, and (3) establishing infrastructure for future negative-emission technologies like BECCS. Realizing this potential requires concurrent policy advancements (carbon prices >200 yuan/ton) and technological innovations (50% cost reductions).
- Implementation Requirements and Economic Synergies: The economic viability of CCUS hinges on cross-sectoral value creation, including enhanced oil recovery (increasing yields by 15-20%) and chemical feedstock production. However, large-scale deployment faces dual challenges: (1) infrastructure demands, requiring 10,000+ km of CO₂ pipelines and 50-100 major storage sites by 2050, and (2) market design needs, particularly flexible electricity pricing mechanisms to accommodate capture systems' energy penalties (20-30% plant output). International benchmarks demonstrate that successful CCUS industrialization depends on integrated policy packages combining carbon pricing, capital subsidies (covering 30-50% of retrofit costs), and liability frameworks for long-term storage.
- Transferable Lessons for Coal-Dependent Economies: While this study's methodology provides a reference framework, implementation strategies must adapt to local contexts: (1) Policy instruments should align with governance systems - carbon markets suit market economies (EU), whereas administrative measures may dominate planned economies; (2) Geological potential dictates infrastructure planning, with Australia's sedimentary basins offering 200+ years of storage capacity versus limited options in Japan; (3) Energy transition speeds determine CCUS relevance - Germany's rapid renewables expansion (50% by 2030) reduces coal dependence faster than India's; (4) Economic thresholds vary, where <$100/ton carbon prices or <4,000 operating hours jeopardize project bankability. China's experience underscores that CCUS effectiveness scales with policy coordination, particularly in synchronizing carbon pricing with grid modernization investments.

3. Can the paper's findings be applied to other countries with high reliance on coal-fired power, or are they specific to China's energy policies and conditions?

- The author has added the following contents in the discussion part.
- Technological and Policy Gaps in CCUS Development: Significant disparities exist between China and developed nations in CCUS technology maturity, economic viability, and policy frameworks. While countries like those in the EU and U.S. have established commercial-scale operations through integrated technology chains and market-based mechanisms23, China remains in the demonstration phase. For instance, Europe's Northern Lights project and the U.S. Decatur facility achieve million-ton-scale annual capture, whereas China's flagship Guoneng Taizhou project currently operates at 0.5 million tons/year capacity. These gaps stem from differences in both technological readiness and policy approaches - developed economies leverage carbon markets (e.g., EU ETS) and tax incentives (e.g., $85/ton under the U.S. Inflation Reduction Act), while China predominantly relies on direct fiscal subsidies without robust market drivers.

4. What are the key differences between the low-proportion and high-proportion carbon capture installation scenarios?

- Low-proportion carbon capture installation scenario, with an installation proportion of 30%; high-proportion carbon capture installation scenario, with an installation proportion of 90%.

5. How does the paper predict the investment required for carbon capture technology in the coal power sector by 2050, and what are the estimated costs?

- The author has added the following contents in the conclusion part.
- The environmental impacts of CCUS deployment present both benefits and challenges. A key benefit is the potential for near-zero emissions from coal-fired power plants, with capture rates reaching 90% and reducing annual CO₂ emissions by approximately 2 million tons per unit. Additionally, CCUS systems can simultaneously decrease sulfur and nitrogen oxide emissions by 30-50%, contributing to improved air quality. The sequestration of CO₂ also plays a crucial role in mitigating long-term climate risks and supporting carbon neutrality objectives. However, these benefits come with potential costs: CCUS implementation increases system energy consumption by 20-30%, and reliance on coal plants for energy replenishment may temporarily raise grid carbon emissions by 5-8%. Furthermore, the degradation of chemical absorbents could generate harmful byproducts like nitrosamines, necessitating stringent regulatory oversight.

6. How might the deployment of carbon capture technology in coal-fired power plants impact China's energy market and economy?

- The author has added the following contents in the introduction part.
- The economic and environmental trade-offs of CCUS evolve over time. In the short term (pre-2030), high emission reduction costs (300-600 yuan/ton) result in negative net environmental benefits, requiring substantial policy subsidies (covering ~60% of costs). However, long-term projections (post-2040) indicate a shift toward synergy: technological advancements are expected to reduce costs to 150 yuan/ton, while grid decarbonization will lower lifecycle emissions by over 85%. This transition could align with the development of a 20 trillion yuan industrial sector, creating a sustainable balance between economic growth and emission reductions.
- To reconcile early-stage challenges, a three-pronged strategy—combining green electricity capture, pipeline infrastructure, and carbon pricing—is essential. Analysis shows that under high deployment (90%), cumulative emission reductions (2.9 billion tons by 2050) can offset transformation costs. However, achieving this hinges on consistent policy support, particularly carbon pricing exceeding 200 yuan/ton, to ensure economic viability and environmental effectiveness.

7. What are the technological challenges and policy considerations highlighted by the authors for implementing large-scale carbon capture in China?

- 5.7 The role of CCUS technology in achieving China's "dual carbon" goals
- Strategic Importance of CCUS for China's Decarbonization: CCUS technology represents a critical pathway for China to achieve its carbon peaking (2030) and neutrality (2060) targets, particularly for hard-to-abate sectors like coal-fired power. Projections indicate it could deliver 15-32% of cumulative emission reductions, with high-deployment scenarios potentially mitigating 1.2-2.9 billion tons of CO₂ annually by 2050. Beyond emissions mitigation, CCUS supports three key transition objectives: (1) ensuring energy security through reliable low-carbon baseload power during renewable integration, (2) catalyzing a 20+ trillion yuan industrial chain with associated green employment, and (3) establishing infrastructure for future negative-emission technologies like BECCS. Realizing this potential requires concurrent policy advancements (carbon prices >200 yuan/ton) and technological innovations (50% cost reductions).
- Implementation Requirements and Economic Synergies: The economic viability of CCUS hinges on cross-sectoral value creation, including enhanced oil recovery (increasing yields by 15-20%) and chemical feedstock production. However, large-scale deployment faces dual challenges: (1) infrastructure demands, requiring 10,000+ km of CO₂ pipelines and 50-100 major storage sites by 2050, and (2) market design needs, particularly flexible electricity pricing mechanisms to accommodate capture systems' energy penalties (20-30% plant output). International benchmarks demonstrate that successful CCUS industrialization depends on integrated policy packages combining carbon pricing, capital subsidies (covering 30-50% of retrofit costs), and liability frameworks for long-term storage.
- Transferable Lessons for Coal-Dependent Economies: While this study's methodology provides a reference framework, implementation strategies must adapt to local contexts: (1) Policy instruments should align with governance systems - carbon markets suit market economies (EU), whereas administrative measures may dominate planned economies; (2) Geological potential dictates infrastructure planning, with Australia's sedimentary basins offering 200+ years of storage capacity versus limited options in Japan; (3) Energy transition speeds determine CCUS relevance - Germany's rapid renewables expansion (50% by 2030) reduces coal dependence faster than India's; (4) Economic thresholds vary, where <$100/ton carbon prices or <4,000 operating hours jeopardize project bankability. China's experience underscores that CCUS effectiveness scales with policy coordination, particularly in synchronizing carbon pricing with grid modernization investments.

8. What are the environmental implications of carbon capture technology deployment in coal-fired power plants, and how do these compare with the economic benefits outlined in the paper?

- The author has added the following contents in the discussion part.
- 5.8 The environmental impact and economic benefits of deploying carbon capture technology (CCUS) in coal-fired power plants exhibit phased characteristics
- The technical challenges of implementing CCUS in China's coal-fired power sector are multifaceted. Firstly, the cost bottleneck remains a critical issue, as post-combustion capture technology currently reduces power plant efficiency by 15%-25% and incurs high unit costs of 300-600 yuan/ton CO₂. Addressing this requires breakthroughs in key technologies such as advanced adsorbents to achieve cost reductions. Secondly, scaling presents a significant challenge, with existing demonstration projects capturing less than one million tons annually, while billion-ton deployments will be necessary by 2050. This gap underscores the urgent need to resolve engineering stability issues during large-scale implementation.
- Key policy considerations must focus on creating an enabling environment for CCUS deployment. Establishing comprehensive economic incentives through a "carbon pricing + subsidies + green finance" policy framework is essential, with carbon prices needing to exceed 200 yuan/ton to ensure viability. Infrastructure development should be prioritized, particularly the construction of a North China-Northwest CO₂ transportation pipeline network to reduce marginal costs. Additionally, regulatory frameworks must be strengthened to clarify long-term storage responsibilities and resolve property rights disputes, providing the legal certainty required for large-scale investments. These measures collectively address both the technical and systemic barriers to CCUS implementation in China's energy transition.

9. Literature review/ introduction section needs to be improved significantly. All the references are fairly old. It is suggested to add some recent reference related to this work, from 2023-2024.

- The author has improved the introduction part, and added the published work in 2023-2024.

Reviewer #4: The manuscript presents a comprehensive assessment of carbon capture technology deployment in China’s coal-fired power sector. The study evaluates various scenarios, predicting carbon emissions, capture potential, and investment needs before 2050. The topic is relevant given global carbon neutrality goals, and the manuscript is structured with a clear methodology, detailed scenario analysis, and quantitative predictions. However, some areas require clarification and improvement to enhance the study’s robustness.

Text: "The paper also predicts and analyzes the installation cost, operating cost, and investment scale of coal-fired carbon capture facilities before 2050 under seven scenarios." (Page 1)
→ Consider explicitly stating the key assumptions behind cost calculations to improve transparency.

- The author has added the following contents in the introduction.
- This paper establishes three key assumptions. First, a technical assumption specifies that coal-fired carbon capture, utilization, and storage (CCUS) primarily employs post-combustion capture, with costs and efficiency following current trends (e.g., an emission factor of 0.899 kg/kWh). Second, a policy assumption posits that seven scenarios—such as policy reinforcement and temperature control targets—can encompass China’s potential emission reduction pathways. Third, an economic assumption projects that carbon capture investment and operating costs can be extrapolated to 2050 based on existing data. Unlike prior studies focusing on singular technological or economic analyses, this paper employs multi-scenario modeling to systematically forecast the large-scale deployment needs of coal-fired CCUS in China under 1.5°C/2°C targets. By integrating International Energy Agency (IEA) projections with Chinese policy scenarios, this study reveals the potential scale of an emerging CCUS-driven industry, offering novel insights for policymaking.

Text: "CCUS technology is a key technology for achieving low-carbon utilization of fossil fuels and supporting low-carbon emissions in industrial processes such as coal-fired power." (Page 1)
→ Clarify whether the study focuses on specific CCUS technologies (e.g., post-combustion, pre-combustion) or a general assessment.

- The author has added the following contents in the introduction.
- While prior studies address techno-economic aspects of CCUS, systematic projections of deployment pathways, investments, and emission reductions under diverse climate targets remain scarce. This study fills this gap by quantifying CCUS capacity, costs, and mitigation potential across the seven scenarios. Using pre-2050 power system data (installed capacity, generation, and coal-fired share), we project coal-power emissions via a 0.899 kg/kWh emission factor (Shanghai Standard DB31/T 1139-2019). We further estimate 2050 installation/operation costs and annual investments (2030–2050) for low- and high-penetration CCUS, revealing cumulative costs under both scenarios. Results demonstrate that high-penetration CCUS can drive coal-power decarbonization, with investments potentially exceeding 20 trillion yuan, fostering a new green industry while balancing low-carbon transition and economic growth.

Text: "The International Energy Agency (IEA) also estimates that the total amount of CO2 captured using carbon capture technology globally will increase from approximately 40 million tons in 2020 to approximately 7.6 billion tons by 2050." (Page 2)
→ Cite the specific IEA report and year for accurate reference.

- The International Energy Agency (IEA)'s "Net Zero by 2050: A Roadmap for the Global Energy Sector" (2021) also assesses that the total amount of CO2 captured using carbon capture technology globally will increase from approximately 40 million tons in 2020 to approximately 7.6 billion tons in 2050, representing a more than one hundred-fold increase.

Text: "According to different engineering techniques, it can be divided into CO2 geological utilization, CO2 chemical utilization, and CO2 biological utilization." (Page 3)
→ Provide specific examples or case studies for each category to improve clarity.

- The author has added the following contents in the introduction.
- Geological Utilization Pathways: Geological applications demonstrate significant scale potential, with two primary methods: Enhanced Oil Recovery (EOR), as implemented in China's Jilin Oilfield project (300,000 tons CO₂/year stored with 15% oil yield increase), and deep saline aquifer storage exemplified by Norway's Northern Lights project (current 1.5 million tons/year capacity, expanding to 5 million). These methods provide immediate emission reductions while creating economic value through either hydrocarbon production or secure long-term sequestration.
- Chemical Conversion Applications: Chemical utilization transforms CO₂ into commercial products through catalytic processes. Notable examples include Iceland's geothermal-powered methanol synthesis (4,000 tons/year) and China's Ningxia coal-to-urea project (500,000 tons CO₂/year). Advanced materials production is demonstrated by Germany's Covestro, manufacturing polycarbonates with 0.5 tons CO₂ fixed per ton product. These applications showcase the potential for carbon circularity in chemical manufacturing sectors.
- Biological Utilization Methods: Biological pathways offer dual environmental benefits through microalgae cultivation (e.g., U.S.-based Algenol's 200 tons CO₂/hectare/year biofuel production) and agricultural enhancement (Dutch greenhouse operations achieving 20-30% yield increases at 800-1000 ppm CO₂). These nature-based solutions complement engineered approaches while supporting food and energy production systems.
- Storage refers to the permanent sequestration of CO₂ in deep geological formations, isolating it from the atmosphere. As the only technology enabling low-carbon fossil fuel use and large-scale emissions reduction, CCUS plays a critical role in climate mitigation strategies[7].

Text: "This article focuses on studying the carbon capture capacity, carbon reduction potential, and investment demand prediction of coal-fired power generation." (Page 3)
→ Explain how these predictions compare with international benchmarks.

- The author has added the following contents in the introduction.
- Coal-fired CCUS technology captures and purifies CO₂ emissions from power plants for subsequent utilization or permanent storage, with post-combustion capture currently dominating engineering applications. While utilization and storage pathways remain diverse with uncertain market adoption, this study focuses on system-level assessment of three critical dimensions: (1) capture capacity, (2) emission reduction potential, and (3) investment requirements. Projections indicate China's coal-power CCUS could achieve 2.9 billion tons annual CO₂ reduction by 2050 - representing over 40% of global capacity (IEA's 3 billion ton estimate) - with retrofit costs ($1,000-3,000/kW) being 15-20% lower than Western benchmarks. However, economic viability requires substantial policy support, particularly carbon pricing exceeding 200 yuan/ton, alongside solutions for grid integration challenges arising from capture systems' 20-30% energy penalty.
- Cost Structures and Research Gaps: Existing studies analyze coal-CCUS through three lenses: technological development, economic feasibility, and environmental impacts. The comprehensive cost structure comprises8: (1) economic costs (capital expenditures: $1,000-3,000/kW installation; operational expenses: capture, transport, storage); and (2) environmental costs (15-30% energy penalty; 0.1-0.5% annual leakage risk)9. Current research highlights critical challenges: capture phase dominates system costs (60-75%), efficiency losses may offset 15-25% of emission reductions, and storage site availability limits regional deployment. These factors underscore the need for integrated assessments balancing technological potential with real-world constraints in China's energy transition context.

Text: "Table 1. Carbon capture emissions from coal-fired power in China under seven scenarios by 2050" (Page 5)
→ Some numerical values lack explanations. Consider adding a brief discussion on the variations between scenarios.

- The author has added the following contents in the introduction.
- This study employs seven distinct scenarios to analyze China's coal-fired power CCUS development pathways[10-13]: （1）Policy Scenario: Maintains current low-carbon transformation trends aligned with China's Nationally Determined Contributions (NDCs) under the Paris Agreement. (2) Strengthened Policy Scenario: Builds upon Policy Scenario with Strengthened GDP energy intensity reductions (30-35% below 2005 levels by 2030), and increased non-fossil energy share (>25% primary energy by 2030), with tighter CO₂ emission controls. (3) 2°C Scenario: Models pathways consistent with limiting global warming to 2°C, including deep decarbonization strategies, technology deployment roadmaps, and policy and financial requirements. (4) 1.5°C Scenario: Analyzes net-zero CO₂ pathways by mid-century, assessing: Technical feasibility, Socioeconomic impacts,Greenhouse gas reduction potentials. (5) Literature-Based Scenario[12]: Incorporates projections from China's Energy and Electricity Development Plan (2030-2060). (6) Stated Policies Scenario (SPS): Reflects current policy trajectories based on Global energy market trends, Technology cost projections, Existing national policy frameworks. (7) Announced Pledges Scenario (APS): Assumes full implementation of all national on Climate commitments, Net-zero targets, Energy transition plans
- To predict CO₂ emissions from coal-fired power generation units, this study adopts an advanced benchmarking approach based on the local standard Carbon Emission Indicators for Coal-Fired Power Generation Enterprises (DB31/T 1139-2019). Specifically, we apply the standard’s upper-bound emission factor of 899 g CO₂/kWh to model sector-wide emissions, ensuring alignment with stringent regional performance benchmarks. This method provides a consistent and replicable framework for assessing emission trajectories under varying operational scenarios.

Carbon emissions=power generation * 0.899 (kg/kWh)

Text: "Based on this data, calculate the installation cost of carbon capture for coal-fired power in China under seven scenarios in 2050." (Page 7)
→ Clarify the methodology used for cost estimation (e.g., exchange rate assumptions, inflation considerations).

- The exchange rate between the US dollar and the RMB is calculated at 6.849, without considering inflation. All calculations are based on constant prices in 2025, and the final result is presented in billions of RMB.

Text: "Table 4. Operating costs for carbon capture and emission reduction in China's coal-fired power plants under 7 scenarios in 2050" (Page 9)
→ The operating cost range is wide. It would be useful to explain the key cost drivers leading to this variation.

- The author has added the following contents
- The projected operating costs of CCUS in China’s coal-fired power plants (30–150 yuan/ton CO₂ by 2050) exhibit significant variability due to four key factors: (1) Technical route—post-combustion capture (80–150 yuan/ton) incurs the highest costs due to energy-intensive amine solvent regeneration, while pre-combustion capture (30–50 yuan/ton) benefits from coal gasification synergies, and oxy-fuel combustion (90–150 yuan/ton) depends on air separation unit efficiency. (2) Scale effects—under high adoption (90% deployment), economies of scale could reduce unit costs by ~35%. (3) Energy price sensitivity—a 0.1 yuan/kWh electricity price increase raises operating costs by ~15 yuan/ton. (4) Technological advancement—emerging solvents (e.g., phase-change absorbers) may cut regeneration energy use by 20–30%, though their commercial viability remains uncertain.

Text: "Reference [13] points out that China will promote CCUS technology on a large scale from 2050 to 2060, until it covers all coal-fired power units." (Page 10)
→ Justify why large-scale adoption is expected to occur after 2050 and not earlier.

- The author has added the following contents
- China's coal-fired power fleet totaled 1.08 billion kilowatts by end-2020, comprising subcritical (356 MW, 32.9%), supercritical (285 MW, 26.4%), ultra-supercritical (250 MW, 23.1%), and substandard subcritical units (190 MW, 17.6%)[17]. With over half of operational units constructed during 2005-2015 and assuming 30-year lifespans, the sector will face peak retrofit demands between 2035-2045. Current projections indicate a phased technology transition: first-generation capture systems will dominate retrofits of existing plants before 2035, while second-generation systems will be deployed in new builds thereafter[21]. This aligns with China's power sector roadmap - coal capacity is expected to peak by 2025 under the 14th Five-Year Plan, with no new coal projects post-2030 and gradual retirement of aging units[21,22]. Carbon capture deployment is anticipated to scale from R&D-focused pre-2030 efforts to commercial implementation at 14 GW/year (280 GW cumulative by 2050), contingent on post-peak emission policies[22].
- Text: "The widespread deployment of coal-fired carbon capture technology may create an emerging industry with a scale of over 20 trillion yuan, achieving a win-win situation for the low-carbon transformation of coal-fired power and green economic growth." (Page 13)
  → Consider discussing potential policy challenges that could hinder this large-scale adoption.
- The author has added the following contents
- 5.8 Core challenges and policy considerations for implementing large-scale CCUS in China
- The technical challenges of implementing CCUS in China's coal-fired power sector are multifaceted. Firstly, the cost bottleneck remains a critical issue, as post-combustion capture technology currently reduces power plant efficiency by 15%-25% and incurs high unit costs of 300-600 yuan/ton CO₂. Addressing this requires breakthroughs in key technologies such as advanced adsorbents to achieve cost reductions. Secondly, scaling presents a significant challenge, with existing demonstration projects capturing less than one million tons annually, while billion-ton deployments will be necessary by 2050. This gap underscores the urgent need to resolve engineering stability issues during large-scale implementation.
- Key policy considerations must focus on creating an enabling environment for CCUS deployment. Establishing comprehensive economic incentives through a "carbon pricing + subsidies + green finance" policy framework is essential, with carbon prices needing to exceed 200 yuan/ton to ensure viability. Infrastructure development should be prioritized, particularly the construction of a North China-Northwest CO₂ transportation pipeline network to reduce marginal costs. Additionally, regulatory frameworks must be strengthened to clarify long-term storage responsibilities and resolve property rights disputes, providing the legal certainty required for large-scale investments. These measures collectively address both the technical and systemic barriers to CCUS implementation in China's energy transition.
